# New Dispensaries

**Published:** 1878-02

**Authors:** 


### EDITORIAL.

### New Dispensaries.

We have opportunity to announce the incorporation of a much needed charity (?) "The Children's Medical and Surgical Relief Association." The *advertisement* in the daily papers is as follows:

"A NEW CHARITABLE SOCIETY.

"A new charitable society has been incorporated in this city, to be known as 'The Children's Medical and Surgical Relief Association.' Its aims and objects are explained in the title, its special work being to provide medical and surgical assistance for poor children whose parents are unable to thus properly care for them.

"The following gentlemen have been elected officers of the Association: President, O. H. Marshall; Vice-President, J. P. Dudley; Secretary and Treasurer, Isaac G. Jenkins; Consulting Physicians and Surgeons, James P. White,

M. D., Thos. F. Rochester, M. D.; Physician and Surgeon in charge, Joseph Fowler, M. D.

"Rooms have been established over No. 303 Main street, and they will be open for the reception of patients at three o'clock every day. It is earnestly desired that those who recommend patients will, as far as possible, furnish evidence of their destitute condition. The objects of the society appeal strongly to our charitably disposed citizens. Infantile diseases need careful and delicate treatment, and much suffering is often caused to the unfortunate little ones whose parents are too poor to hire a doctor or get them proper medicine. Furthermore, there are many acute cases of infantile ailment for which it is necessary to secure elsewhere the care and attention which can not be furnished in the homes of the poor. This society will, therefore, undoubtedly fill a want long felt, and may eventually result in the establishment of a children's hospital."

Dr. Chenoweth, in an address before the Illinois Central Medical Society, (extracts from which are in this Journal,) in speaking of the organization by physicians of the various so-called charities, and advertising through them, pays these institutions a most deserved compliment, in furnishing physicians an opportunity of doing exactly what the Code of Ethics forbids, viz.: advertising themselves through the daily papers. He says:

"To the Code we owe the peculiar quackery which belongs to the medical profession—unlike that of other professions, it is not a mere annoying excrescence, but a veritable cancer, not always to be found without search, but ever malignant. While in other professions it is like the dust that covered Egypt, in ours it is like the lice called forth by the wrath of God. We have no hope of its immediate extermination. 'The mortality of man is the salvation of truth,' and this generation, at least, must die before the wiseacres, who control the interests of medicine, discover that physicians are amenable to the same physical, mental and ethical laws as are the balance of mankind.

"In the near future we hope that possession of a diploma will be a guarantee of ability and that no man can obtain it without knowledge. Then the advertisement needed will be of the particular subjects the physician is willing, or best able, to treat.

"I wish to declare distinctly that I have no affinity or sympathy with so-called Medical or Surgical Infirmaries or Institutes, which I regard as swindling concerns not morally superior to three-card monte or bunco banks; and he who sends patients to parties running such institutions is aiding and abetting the enemies of morals and medical science."

We are indebted to a physician of this city for a suggestion which may be of interest to some of our medical friends, and prove of practical value. He says "that it might be well to establish some free dispensaries in the outskirts of the city. That the physicians now have little to do and no pay, that they might thus obtain more work and *some* compensation." In this connection we will suggest that there is an open field for charity, there being, as yet, no free Venereal or Syphilitic Dispensary or *Institution* in Buffalo. Such a field for observation cannot long be neglected with impunity, though it must be acknowledged that the present hospitals and dispensaries monopolize nearly the entire trade, making it a *specialty*, and in some of them an almost exclusive specialty. "Don't do anything else," as the craft say of themselves.

